# Shift of the Muscular Inhibition Latency during On-Line Acquisition of Anticipatory Postural Adjustments

**DOI:** 10.1371/journal.pone.0154775

**Published:** 2016-05-18

**Authors:** Fanny Barlaam, Marianne Vaugoyeau, Carole Fortin, Christine Assaiante, Christina Schmitz

**Affiliations:** 1 Aix-Marseille University, CNRS, UMR 7291: Laboratory of Cognitive Neuroscience, Marseille, France; 2 Fédération de Recherche N3512, Comportement, Cerveau, Cognition, Marseille, France; 3 Lyon Neuroscience Research Center, Brain Dynamics and Cognition Team, CRNL, INSERM U1028, CNRS UMR5292, Lyon, France; 4 University Lyon 1, Lyon, France; Duke University, UNITED STATES

## Abstract

During action, Anticipatory Postural Adjustments (APAs) cancel the consequences of a movement on postural stabilization. Their muscular expression is characterized by early changes in the activity of the postural muscles, before the movement begins. To explore the mechanisms enabling the acquisition of APAs, a learning paradigm was designed in which the voluntary lifting of a load with one hand triggered the unloading of another load suspended below the contralateral forearm. The aim of this study was to investigate changes in the muscular expression that uncovers the progressive learning of new APAs. A trial-by-trial analysis of kinematic and electromyographic signals recorded on the right arm was conducted in twelve adults through six sessions of learning. Kinematic results reported an enhancement of the postural stabilization across learning. The main EMG pattern found during learning consisted of a flexor inhibition, where latency was shifted towards an earlier occurrence in parallel with the improvement of the postural performance. A linear regression analysis conducted between the inhibition latency and the maximal amplitude of elbow rotation showed that the earlier the inhibition onset, the better the postural stabilization. This study revealed that the progressive shift of the postural flexor inhibition latency could be considered as a reliable neurophysiological marker of the progressive learning of new APAs. Importantly, this marker could be used to track motor learning abnormalities in pathology. We relate our findings to the update of a forward predictive model of action, defined as a system that predicts beforehand the consequences of the action on posture.

## Introduction

Anticipatory postural adjustments (APAs) prevent the forthcoming disturbance of posture by cancelling the destabilizing effect of movement on posture [1,2]. APAs characteristics have been explored using the bimanual load-lifting task in which subjects lift with their right hand a load placed on their left postural forearm [3]. In this situation, APAs enable the stabilization of the postural forearm thanks to an inhibition of the flexor muscles before the unloading onset. On the contrary, when the experimenter unexpectedly lifts the load, an upward rotation of the forearm signals the absence of any possible anticipation. This is also known as the “waiter effect” [3].

Across development, the acquisition of anticipatory control is characterized by an early emergence of APAs and a late refinement of the muscular signature sustaining the APAs during childhood [4,5] and adolescence [6]. Their acquisition depends on the previous experience of postural disturbance associated with the movement [4–6]. To explore the physiological mechanisms enabling the formation of APAs, an artificial situation was designed in which the voluntary lifting of the load by the right hand triggers the unloading of the load suspended below the left forearm, which is engaged in a postural stabilization task [7,8]. Although this situation first triggers a postural perturbation, postural stabilization takes over after new APAs are built as a product of trial repetition [7]. Hence, this situation is particularly useful to study the learning processes in play during the formation of APAs.

Motor learning can be defined as the modification of a motor skill performance through practice; it relies on the main role of feedback afferent control for error corrections [9]. Many adaptation paradigms, including visuo-motor adaptation [10], reaching in force field [11,12] and grip force adaptation [13] have been used to study the role of feedback in the context of error-based learning associated to the update of an internal model [14]. Internal models, encompassing inverse and forward models, are considered as systems that mimic the behavior of a natural process [15]. The inverse model transforms the intended outcome of a movement into the appropriate motor commands, whereas forward models predict the next sensory state from the current state and a copy of the motor commands [16]. The ability to quickly update these models could explain the remarkable capacity that humans have to learn a wide range of motor skills [9].

Forward models can also be used to predict adverse consequences of an upcoming action before it occurs [9,17–20]. During the bimanual-load lifting task, the prediction of the consequences of the motor command might rely on a forward predictive model [17,21,22], that could then produce APAs. To date, little information is available on the update of this type of forward model associated with the improvement of motor performance during a learning task.

While the kinematic characteristics of APAs acquisition have been well established in the learning version of the bimanual load-lifting task [7,8,23], few studies have focused on their muscular counterpart. During learning, the progressive formation of APAs could be related to either an increase in postural arm stiffness due to the cocontraction of antagonist muscles [24], or to an early inhibition of the postural muscles. Studies that have evaluated patterns of muscular activity in the early stages of learning generally report that there is a decrease in the cocontraction pattern as learning progresses [12,25]. The cocontraction pattern is a dominant strategy used by young children performing the bimanual load-lifting task when the stabilization of their forearm still presents with postural insecurity [4,5]. Moreover, the equilibrium-point hypothesis claims that individuals might learn to compensate for the disturbing effects of unloading by increasing the antagonist muscle stiffness in the very same task [24]. Hence, the cocontraction pattern could be a marker of the ongoing learning process. Alternatively, studies in adults [26] and developmental studies [4–6] have shed some light on the inhibition pattern, whose rate could vary with either learning or experience. Interestingly, the accurate timing of the onset of muscular inhibition has been found to be a key player in the fine adjustment of APAs [4,6], thus underlying its potential role in learning. Here we sought to clarify which muscular pattern could be associated with the dynamic processes sustaining APAs learning. The aim was to establish a reliable physiological marker of the acquisition of APAs, within a forward predictive model framework.

## Material and Methods

### Participants

Twelve, right-handed, adults aged 23 to 40 years (6 females, mean ± SD age: 29 years ± 5 years 9 month) participated in this experiment. All participants gave their written informed consent before the beginning of the experiment. Local ethics committee approval (Comité de Protection des Personnes Sud Méditerranée 1) was obtained in accordance with the ethical standards of the Declaration of Helsinki.

### Experimental set-up

**Fig 1:**
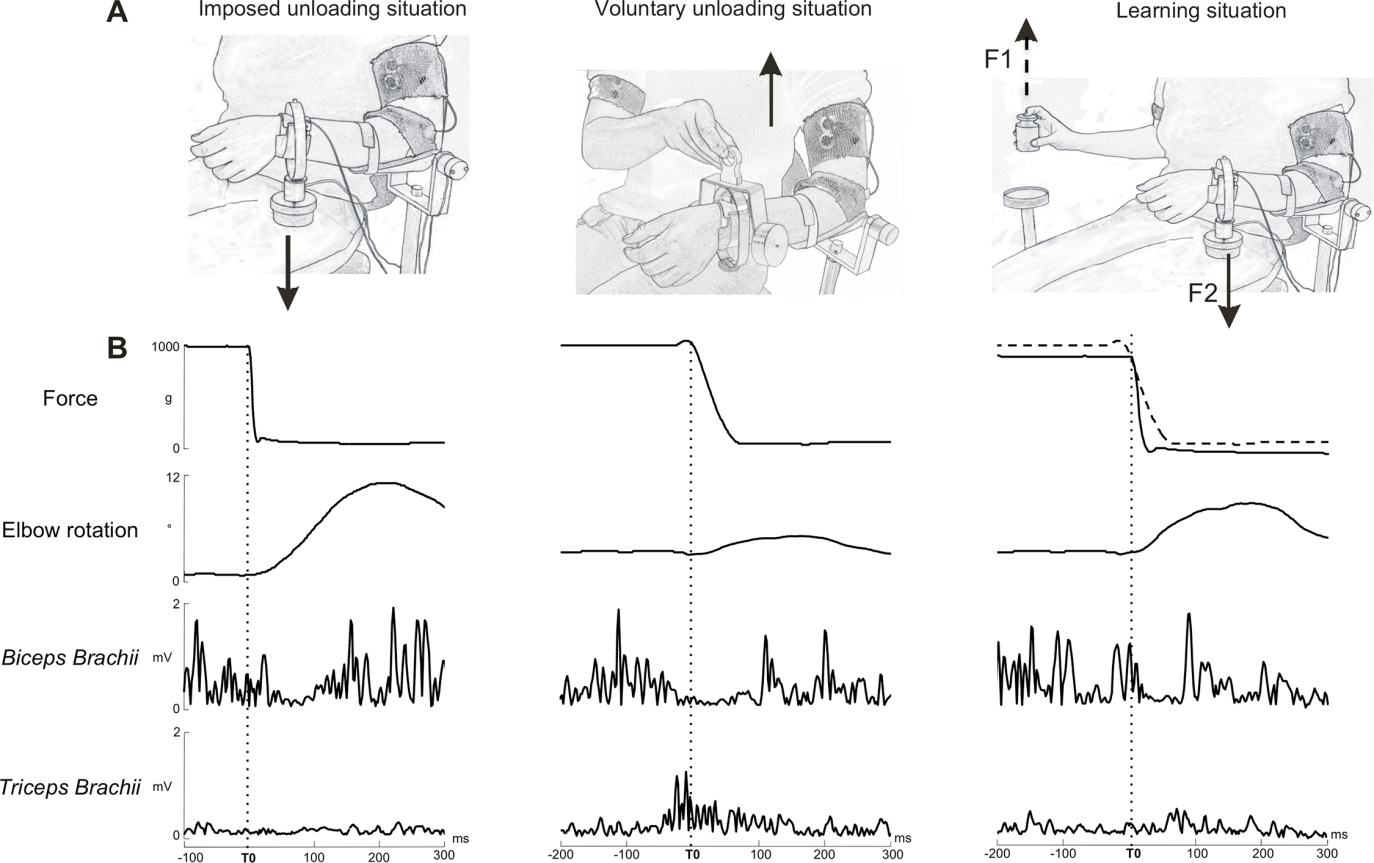
Experimental set-up and raw trial recordings in a subject for each situation. (A, left) Imposed unloading situation: the load was released at unpredictable times by the experimenter. (A, middle) Voluntary unloading situation: the load was lifted by the subject, using the right hand. (A, right) Learning situation: the lifting of the load from the platform by the subject’s right hand triggered the unloading of the load suspended below the left forearm. (B) Parameters recorded are as follows, from top to bottom: force, elbow rotation angle, EMG of the *biceps brachii* and the *triceps brachii* on the postural forearm. The decrease of the force indicated the onset of unloading (vertical line), used as a reference time. During the imposed unloading situation (B, left), note the upward elbow rotation and the decreased activity on the *biceps brachii*, which corresponds to the unloading reflex. During the voluntary unloading situation (B, middle), note the reduced elbow rotation and the early *biceps brachii* inhibition before unloading. During the learning situation (B, right), the force decreased occurring when the subject lifted the object (F1) is depicted by a dashed trace, and is followed by the release of the load suspended below the forearm (F2). The progressive reduction of the elbow rotation amplitude reflects the improvement of the postural stabilization. On this particular trial, a double inhibition pattern was found on the *biceps brachii*. (Reprinted from under a CC BY license, with permission from Elsevier, original copyright 2012).

The experimental set-up of the bimanual load-lifting task has been described in previous papers [6,27]. During the experiment, the postural forearm supports the load while the motor forearm is engaged in lifting the load [2,28]. Participants were comfortably seated on a hardback chair, with the left arm, chosen as the postural one for all subjects, fixed vertically to a support, just above the elbow. Subjects were only asked to maintain their left forearm in a horizontal and semi-prone position throughout the entire session. The left arm was thus engaged in a postural stabilization task, with no specific instructions. The wrist was wrapped with a metallic wristband equipped with a strain gauge. The wristband enabled a load to be either suspended by means of an electromagnet or placed on top of the forearm. On their right side, a similar load was placed on a platform equipped with a second strain gauge. All subjects underwent three situations depicted in Fig 1: first, the imposed unloading situation, then the voluntary unloading situation, and lastly the learning situation. During the imposed unloading situation, the load suspended under the left forearm was suddenly released by the experimenter who switched off the magnet at unpredictable times (Fig 1A left).

The imposed unloading situation was used as a control situation in order to measure the maximal upward forearm displacement resulting from the postural perturbation, and the onset of the unloading reflex. The voluntary unloading situation consisted of participants lifting with their right hand the load placed on top of the left wristband (Fig 1A middle). The learning situation consisted of a succession of trials in which subjects lifted a load placed on a platform situated on their right side, using their right hand. The lifting triggered, via an electronic circuit, the passive unloading of the load below the left forearm (Fig 1A right). Specifically, a 5% decrease in the weight of the right load during its lift from the platform triggered the release of the load suspended below the left postural forearm. A constant 20 ms delay occurred between the onset of the right load lifting and the sudden release of the load below the left forearm. In the voluntary unloading and in the learning situations, subjects were instructed to lift the load immediately upon the experimenter's verbal command.

The experiment was monitored using the Windelest® software developed by TechnoConcept (France). The general procedure was as follows: a series of 10 trials in the imposed unloading situation (pre-IMP), a series of 10 trials in the voluntary unloading situation, 6 series of 10 trials in the learning situation, and a series of 5 trials in the imposed unloading situation (post-IMP). As the repetition of unpredictable perturbations produces a gain in the unloading reflex [3], an additional series in the imposed unloading situation was performed after the learning series to ensure that the latter did not alter the unloading reflex.

In the imposed unloading situation, the release of the load induces an upward elbow rotation whose angular amplitude depends on the forearm length and on the load’s weight. The weight of the load was adjusted for each subject to ensure that measures of the maximal amplitude were within the same range for all participants. We used training trials in the imposed unloading situation to calibrate the weight such that the maximal amplitude of the upward rotation was comprised between 10° and 14°. Thus, the load’s weight was maintained between 800 g and 1000 g. For a given participant, the same weight was used during the three situations. A two minutes rest period was given between each series during the learning situation. An entire session usually lasted around one hour.

### Kinematic, force and electromyographic acquisition

The change of force of the load placed on the postural side was measured by a strain gauge fitted to the metallic wristband supporting the weight. A second strain gauge measured the change of force exerted by the load placed on the platform on the right side. A potentiometer placed along the elbow joint axis measured the angular displacement of the forearm. EMG data were collected using bipolar surface electrodes (surface area: 2.5 mm^2^) placed over the surface of two flexors (*biceps brachii* and *brachioradialis*) and one extensor (lateral *triceps brachii*) on the postural upper arm.

EMG, force and angular elbow displacement signals were recorded, digitalized and stored on a computer disk (Windelest®, TechnoConcept, France). EMG signals were amplified with a TELEMG multi-channel electromyograph (BTS). Kinematic and EMG signals were acquired on the same data acquisition card with a 500 Hz sampling-rate.

### Force, kinematic and electromyographic analysis

Each trial was viewed offline on a monitor screen. Measurements were performed with the MatLab software program (The MathWorks, Inc.). Fig 1B illustrates a single trial recorded in the same participant in each experimental situation. The onset of unloading (T0), used as the reference time, was defined as the first deflection of the force signal transmitted by the strain gauge. To determine T0, a semi-automatic method was used. The mean force signal and its standard deviation (SD) were computed for 0 to 450 ms after the beginning of the trial. A horizontal cursor was positioned when the force signal was statistically significant different from the mean– 3 SD. Visual inspection was then used to confirm the goodness of the automatic definition of T0.

The upward movement of the postural forearm was quantified by measuring the maximum angular amplitude (MA) after unloading. To compare the performance between subjects, MA was expressed in percentages (MA%) of the mean value of the MA obtained during the pre-learning imposed unloading (pre-IMP) situation in each subject for each trial.

MA%≡(MAsituationmeanMApreimp)×100

EMG signals were filtered (5–100 Hz band pass) and rectified using the MatLab software program (The MathWorks, Inc). The EMG signals of each muscle were first visually inspected trial-by-trial on a monitor screen. Compared to the tonic activity that enabled the load to be carried by the postural arm, around the unloading, the EMG events consisted of activations characterized by an increase of activity, or inhibitions characterized by an EMG signal nearing zero. Following this visual inspection, one participant, whose EMG traces presented with artifact in the learning situation, had to be discarded. We quantified the presence of activations and/or inhibitions using a semi-automatic algorithm. Activations were defined as a burst that was more than 2 SDs above tonic activity (measured during the stable phase, when the load was supported by the forearm) and that lasted for 50 ms at least. Inhibitions were defined as a minimum of 70% decrease in activity, compared to the tonic activity, lasting at least 50 ms. When the EMG level of activity around unloading did not adhere to any of these criteria, the trial was labeled as non-identifiable. For each subject, this first step resulted in the definition of EMG events per muscle. The two following indexes were then used to characterize the EMG changes in activity [4,6]:

#### 1. Ratio of EMG patterns

To evaluate the repertoire of muscle activity patterns, EMG responses were analyzed for the two pairs of antagonist muscles (*biceps brachii*/*triceps brachii and brachioradialis/triceps brachii*). Three categories of EMG patterns were found: a cocontraction pattern (concomitant bursts in both flexor and extensor muscles), a single inhibition pattern (one inhibition on the flexor) or a double inhibition pattern (two successive inhibitions on the flexor). For each subject, ratios of each category of EMG pattern were calculated by dividing the number of trials obtained in each category by the total number of trials, and was expressed in percentages. The ratio of non-identifiable trials was calculated similarly.

#### 2. Inhibition latency

The inhibition latency was measured on the flexors as the time-interval between the unloading onset and the onset of the decrease in activity. The inhibition latency was determined trial-by-trial using an interactive software program enabling a well-trained examiner to visually determine the onset of the decrease in activity to the nearest millisecond in reference to the baseline EMG activity before unloading. Indeed, when the EMG signal started to decrease, inhibition onset was measured at the moment the EMG activity fell near to zero. Measurements were performed by a unique well-trained researcher who was familiar with this step as she conducted the same detection step in two previous studies (Barlaam et al., 2011 & Barlaam et al., 2012). Further, to ensure reproducibility, inhibition onset detection was performed twice and no difference was found in the test-retest analysis.

As the ratio of cocontractions was negligible (see Results section), cocontraction latency was not reported.

### Statistical analysis

Kinematic analyses were conducted over 10 trials per series in each situation. Conversely, for the EMG analysis, the existence of non-identifiable trials constrained the EMG analysis. Each series contained a minimum of 3, with an average of 5 identifiable trials for each participant.

We first tested if the kinematic variables (MA° and MA%) and EMG measures (inhibition latency) fitted a Gaussian distribution using the D’Agostino and Pearson normality test [29]. When they fitted a Gaussian distribution, parametric tests were used and statistics given in the text and the figures refer to means and standard deviations. Otherwise, non-parametric tests were used (Mann-Whitney test). Differences with a p -value < 0.05 were considered statistically significant.

To investigate the postural performance changes during the learning process, statistical analyses were conducted on the kinematic parameters in the learning situation. MA% was averaged across all the trials in each series. First, to evaluate learning efficiency, a one-sample t-test was used to compare the MA% of the first series to the reference value of 100%, which corresponds to the maximal level of postural perturbation obtained during the imposed unloading situation. Second, the learning effect was analyzed across the series using a repeated measure Analysis of Variance (ANOVA). When a main significant learning effect was found, we applied multiple comparison (i.e. post-hoc) Bonferroni t-tests. Finally, to evaluate the level of postural stabilization at the end of the learning situation, MA% in the last series of the learning situation was compared to MA% in the voluntary unloading situation using a t-test.

To characterize the learning dynamics in each series, a non-linear regression analysis was performed on the MA% using an exponential decay function as it classically describes a learning curve see for review([30]). The R^2^ ratio was used to assess the goodness of fit.

For the EMG analyses, all variables were averaged over the number of identifiable trials for each subject and within each specific pattern. To examine the alteration of the flexor inhibition latency during the learning situation, we compared the inhibition latencies of the first and the last series of the learning situation to the inhibition latencies of the imposed and the voluntary unloading situations using t-tests.

Lastly, we ran a linear regression to investigate the possibility of a relationship between the onset of inhibition and the level of postural stabilization. In each participant, we isolated the trials showing a single inhibition pattern, and for each of these trials we measured the inhibition latency and its associated MA% as continuous variables. Then, we pooled the trials of the six learning series. Linear regression analyses were run for each subject, and individual values of the slope were extracted and compared to a theoretical zero value using a one-sample t-test.

## Results

### Imposed unloading situation

The comparison between the pre-learning and the post-learning series of the imposed unloading situation did not reveal any significant differences for the mean value of the MA expressed in degrees (respectively, 12 ± 5.23° versus 11.59 ± 4.1°). Inhibitions of the flexor muscles, also known as the unloading reflex [3], were the only pattern found in this situation (ratio of non-identifiable and single inhibition patterns: 37,5% and 62,5% respectively). No difference between the pre-learning and the post-learning series was found for the inhibition latency measured over the *biceps brachii* (respectively, 44.92 ± 10.2 ms versus 45.28 ± 9.5 ms) and the *brachioradialis* (respectively, 58.06 ± 7.48 ms versus 57.19 ± 15.74 ms). As there were no kinematic or EMG differences between the pre-learning and post-learning series, we only used the first series of imposed unloading for comparisons with the learning situation.

### Voluntary unloading situation

**Fig 2:**
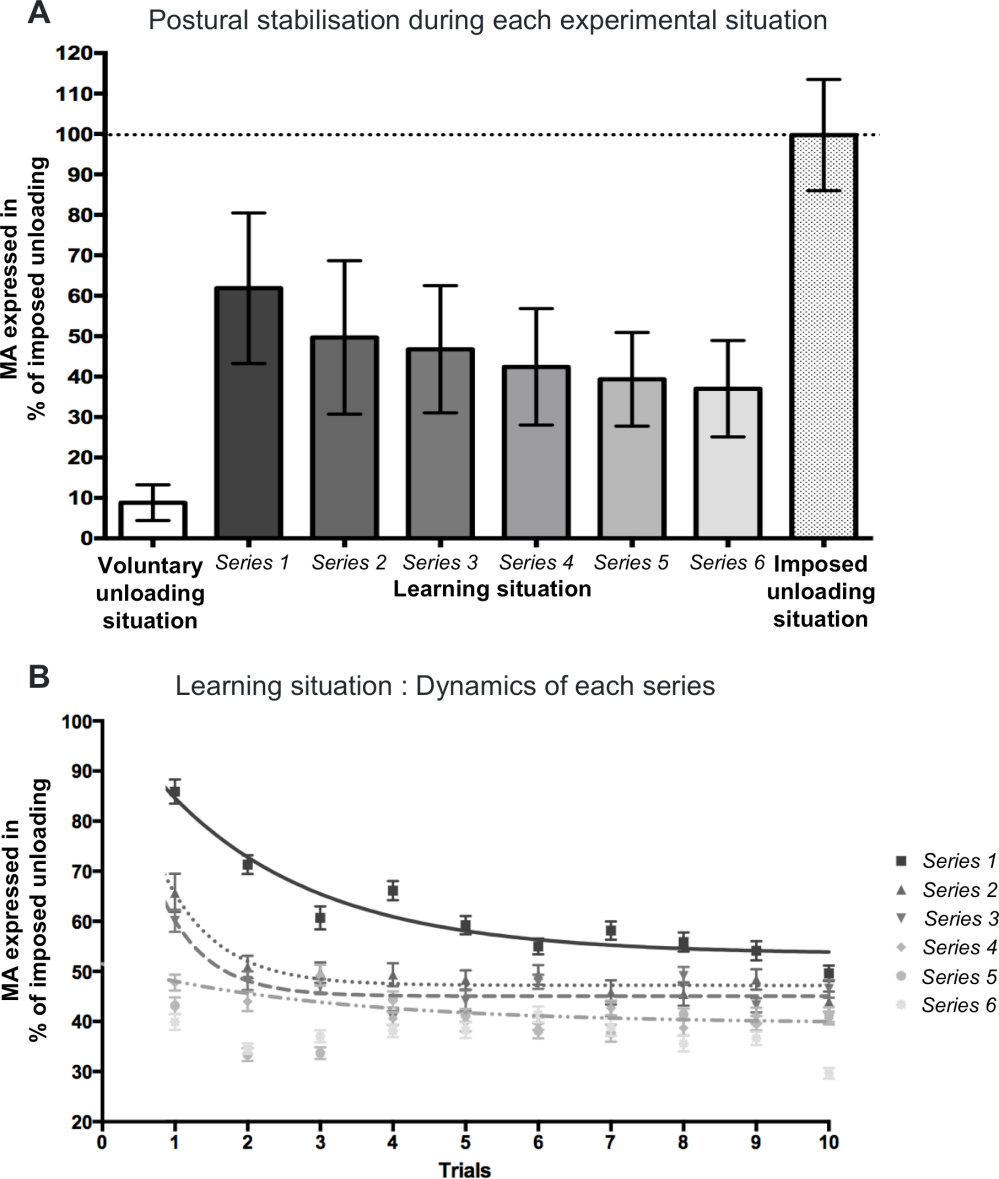
Postural stabilization. (A) For each situation, the maximal amplitude of the upward forearm displacement (MA%) is expressed in percentage of the maximal amplitude measured during the imposed unloading situation. The dotted line indicates the reference value of the imposed unloading situation. Descriptive statistics are expressed as means and standard deviations. (B) Trial-by-trial evolution of the MA% during each series of the learning situation. The variability of the MA% is represented by the Standard Error of the Mean (SEM). A non-linear regression approximates the process of learning during the four first series of the learning situation.

During the voluntary unloading situation, the mean values of MA % (8.82 ± 4.4%, Fig 2A) were significantly reduced (*t*_(1,21)_ = 68.61, *p* < 0.0001) compared to the reference value of 100%, indicating an effective stabilization of the postural arm. In this situation, no participants exhibited a cocontraction pattern. Most of the trials showed a clear single inhibition pattern (ratio of non-identifiable and single inhibition patterns were of 27,68% and 72,32% respectively). Inhibitions over the biceps brachii and the brachioradialis were characterized by an early onset before the unloading (respectively −32.26 + 15.92 ms and −23.65 + 20.59 ms). These results are consistent with previous description of APAs in adults in this task (see for review [2]).

### Learning situation

#### Kinematic analysis

The mean values of MA% of the first series were significantly reduced compared to the reference value of 100% (Table 1, Fig 2A), indicating that effective learning had already taken place.

**Table 1:** MA%, latencies of the single and the double inhibition, and statistical analysis in the learning situation. Descriptive statistics are expressed as means and standard deviations.

		Postural stabilization				Latency of the inhibition measured on the flexor muscle				
		MA %			Single inhibition pattern		Latency 1 in the double inhibition pattern		Latency 2 in the double inhibition pattern	
Learning situation	Value	Comparison to theorical value of 100%	Comparison to voluntary unloading situation	Value	Comparison to imposed unloading situation	Comparison to voluntary unloading situation	Value	Comparison to voluntary unloading situation	Value	Comparison to imposed unloading situation
	(mean ± SD)			(mean ± SD)			(mean ± SD)		(mean ± SD)	
Series 1	61.87 ± 18.60	*t*_(1.21)_ = - 6.79	t_(1.21)_ = 9.74	12.61 ± 37.20	*t*_(1.21)_ = 2.65	*t*_(1.21)_ = - 4.027	"- 76.15 ± 21.69	*t*_(1.21)_ = 4.67	35.85 ± 25.58	NS
		*p < 0*.*0001*	*p < 0*.*0001*		*p* = 0.029	*p* = 0.003		*p* < 0.001		
Series 2	49.69 ± 18.97	-	-	"- 12.86 ± 30.4	-	-	"- 55.83 ± 24.04	-	45.94 ± 19.99	-
Series 3	46.77 ± 15.72	-	-	"-12.72 ± 28.69	-	-	"- 67 ± 12.41	-	47.77 ± 13.11	-
Series 4	42.42 ± 14.40	-	-	"-7.62 ± 27.83	-	-	"- 57.44 ± 14.73	-	39.98 ± 13.11	-
Series 5	39.34 ± 11.58	-	-	"-20.26 ± 34.49	-	-	"- 65.96 ± 8.19	-	43.61 ± 9.44	-
Series 6	37.01 ± 11.91	*t*_(1.21)_ = -17.54	*t*_(1.21)_ = 8.99	"-15.54 ± 24.97	*t*_(1.21)_ = 6.87	NS	"- 69.81 ± 29.02	*t*_(1.21)_ = 3.50	40.97 ± 12.25	NS
		*p* < 0.0001	*p < 0*.*0001*		*p* < 0.001			*p* = 0.003		

A main effect was found in the learning situation (*F*_(5,61)_ = 14.98, *p* < 0.001). Post-hoc test showed that MA% of series 1 was significantly higher than those of the others series (*t*_(1,21)_ = 3.71; *p* = 0.008 for series 2; *t*_(1,21)_ = 4.59, *p* < 0.001 for series 3; *t*_(1,21)_ = 5.92, *p* < 0.001 for series 4; *t*_(1,21)_ = 6.86, *p* < 0.001 for series 5; *t*_(1,21)_ = 7.57, *p* < 0.001 for series 6, respectively). Similarly, MA% of series 2 was significantly higher than those of series 5 (*t*_(1,21)_ = 3.15, *p* = 0.041) and series 6 (*t*_(1,21)_ = 3.86, *p* = 0.05).

A significant difference was found between the MA% mean value in the last series and the voluntary unloading situation, suggesting that at the end of the learning situation, the level of postural stabilization did not reach that of the voluntary unloading situation (Table 1, Fig 2A).

Fig 2B illustrates the trial-by-trial evolution of the elbow rotation during all series of the learning situation. To evaluate the dynamic of learning across trials during each series, a non-linear regression analysis was performed on the basis of an exponential decay function. This non-linear function modeled the acquisition process with good approximation for the four first series (R^2^ = 0.914 for series 1, R^2^ = 0.913 for series 2, R^2^ = 0.77 for series 3, R^2^ = 0.56 for series 4).

#### EMG patterns

**Fig 3:**
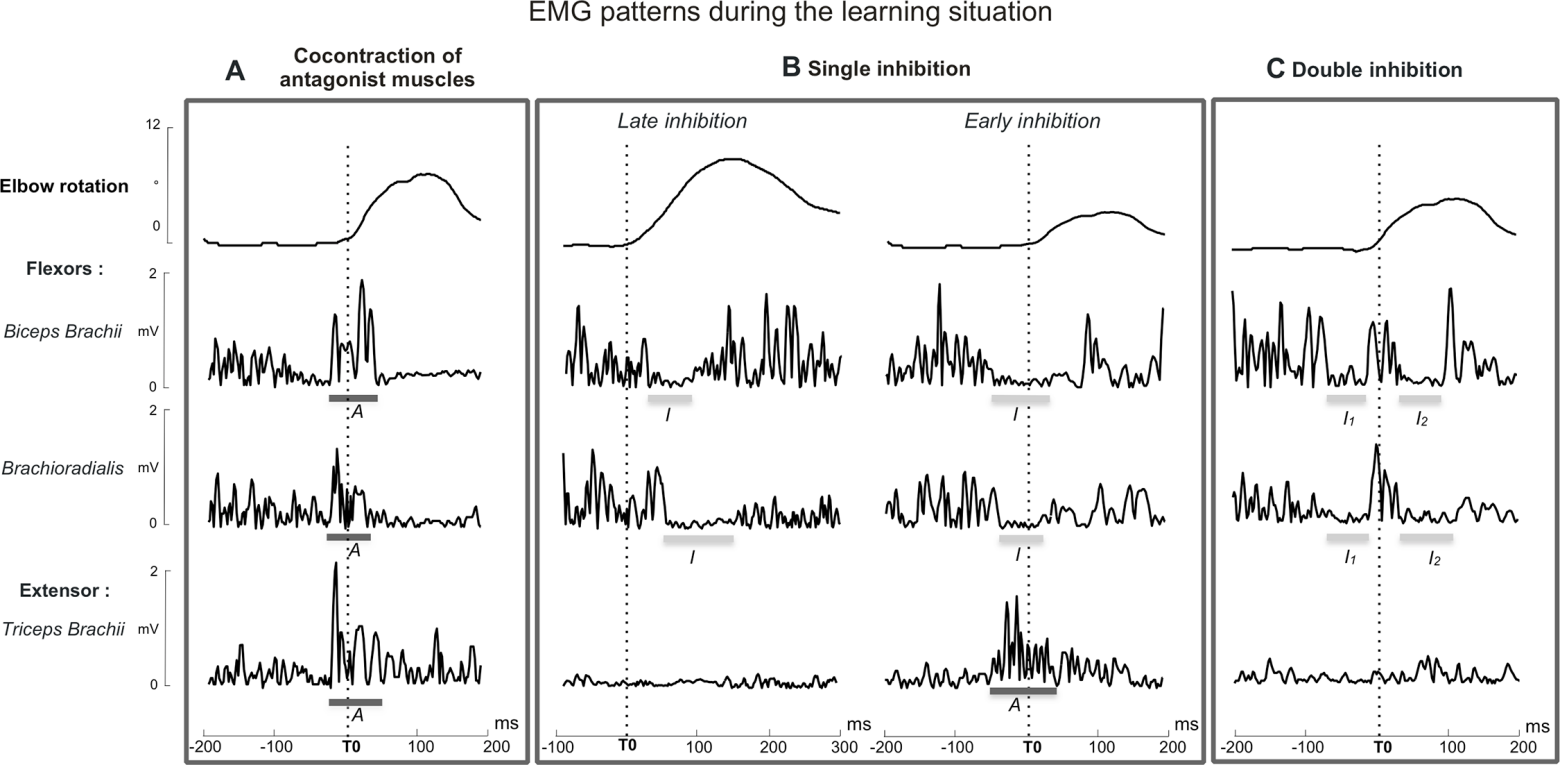
Muscular expression of the forearm stabilization. Three types of EMG responses associated with the elbow rotation recorded on antagonist postural muscles in the same subject during the learning situation. (A) Cocontraction pattern characterized by a simultaneous increase of muscular activity on the flexors and the extensor. (B) Single inhibition pattern characterized by a reduction of activity on the flexors. Note that in these two illustrative examples of the same pattern, the decreased activity could start before or after the unloading onset in the same subject. (C) Double inhibition pattern characterized by two consecutive reductions of activity on the flexors. Black thick lines indicate EMG activity increases (Activation: A). Grey thick lines indicate EMG activity decreases (Inhibition: I). I1 and I2 indicate the first and second inhibitions found in the double inhibition pattern.

Fig 3 reports three types of EMG responses that can be observed during the learning situation. Note that these three examples of different EMG patterns were recorded in the same subject, and thus reveal the co-existence of several muscular patterns during the learning process. As all measurements gave similar results for the *biceps brachii* and the *brachioradialis*, we chose to present the results on the *biceps brachii* only.

The first pattern was characterized by a simultaneous increase in activity between the flexor and the extensor, corresponding to a cocontraction pattern of activity (Fig 3A). The second pattern was characterized by a sudden decrease of the flexor activity, often associated with an increase of the extensor activity. This single inhibition (Fig 3B) started either after the unloading onset (+ 35 ms), similarly to what happens in the imposed unloading situation, or before the unloading onset in the voluntary unloading situation (- 25 ms). In the third type of response, two consecutive inhibitions were clearly recorded over the postural flexor. In this pattern of double inhibition (Fig 3C), the first inhibition over the postural flexor started before the unloading onset (- 60 ms), whereas the second inhibition over the postural flexor started after the unloading onset (+ 40 ms).

Ratios of the different EMG patterns were calculated for the pair of antagonist muscles (*biceps brachii*/*triceps brachii*), and for each series. The main purpose of Table 2 is to illustrate the distribution of the three main patterns (cocontraction pattern, single inhibition and double inhibition) used during the learning situation. The ratio of non-identifiable trials is also reported.

**Table 2:** Ratio of EMG pattern (Cocontraction, single inhibition and double inhibition) and non-identifiable trials during each series of the learning situation.

	Percentages of EMG pattern in biceps brachii/triceps brachii					
	Series 1	Series 2	Series 3	Series 4	Series 5	Series 6
Non-identifiable trials	42.83%	31.82%	39.29%	34.55%	32.02%	28.18%
Cocontraction pattern	0.91%	0.91%	0%	0%	0%	0%
Single inhibition pattern	48.08%	55.45%	46.97%	50%	52.32%	50%
Double inhibition pattern	8.18%	11.82%	13.74%	15.45%	15.66%	21.82%

During the learning process, the ratio of each EMG pattern varied. The cocontraction pattern was only present in the two first series and in very few trials (0.91% for the two series). The ratio of the main pattern of single inhibition was constant regardless of the series of learning (around 50%). The ratio of the double inhibition pattern regularly increased with the repetition of the series (8.18% for series 1 to 21.82% for series 6). The distribution of the EMG patterns was not homogenous through all individuals (see S1 Fig). A portion of the participants did not use the double inhibition pattern, rather they exclusively used the single inhibition pattern. Others mixed these two EMG strategies. While the single inhibition pattern was found in all individuals, no participant presented with the double inhibition pattern alone. Across the learning series, the late inhibition pattern occurred at the beginning of the learning, whereas the early inhibition pattern characterized the EMG pattern of a well-learned behavior.

#### Latency of the inhibition

**Fig 4:**
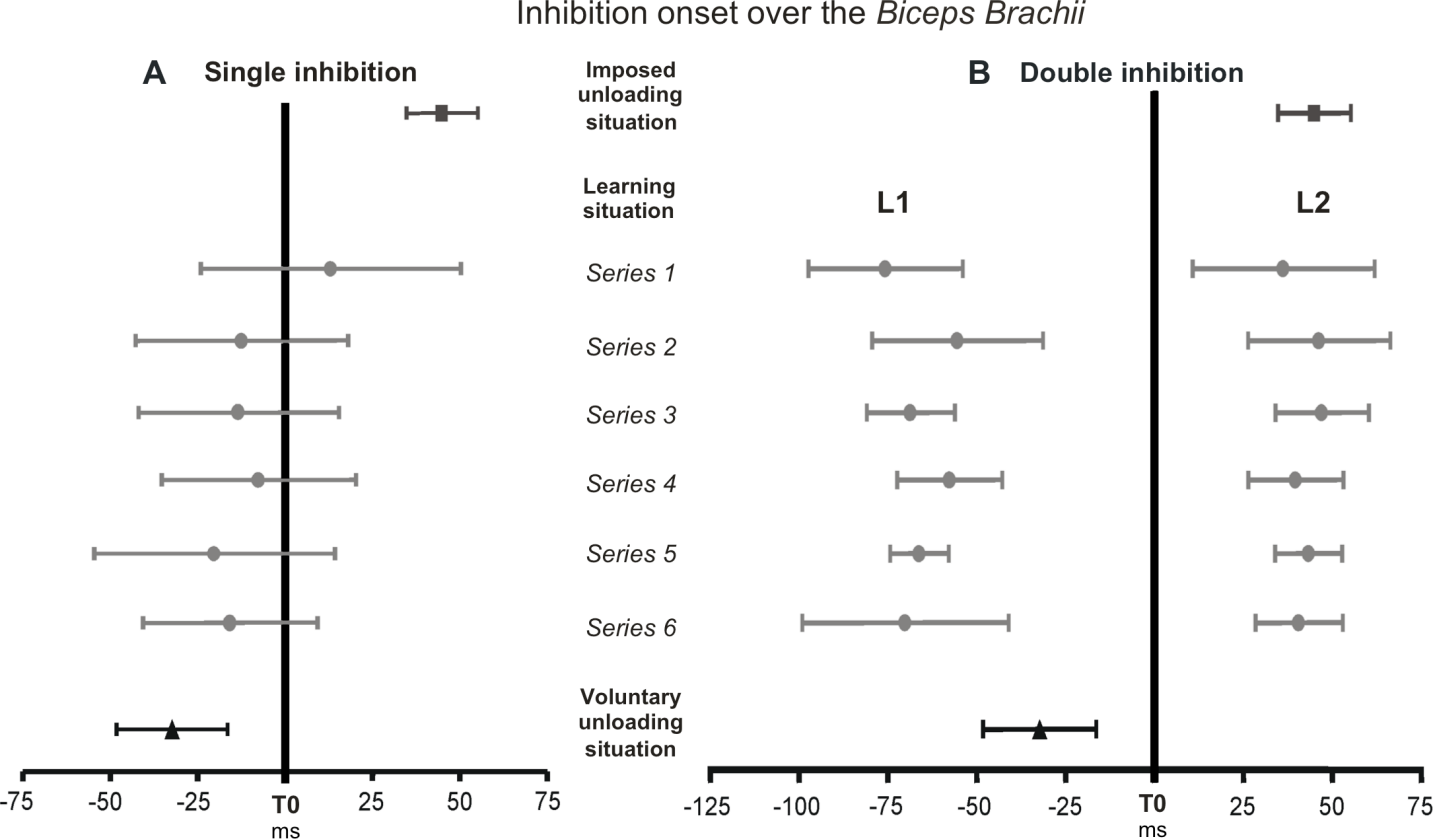
**Onsets of the inhibition over the *biceps brachii* for the single inhibition pattern** (A) and the double inhibition pattern (B) in each situation. In the learning situation, L1 and L2 represent the latencies of the first and the second inhibition during the double inhibition pattern. Descriptive statistics are expressed as means and standard deviations.

Fig 4 represents the latency of the inhibition measured from the biceps brachii for each EMG pattern (part A, the single inhibition; part B, the double inhibition).

The single inhibition latency differed from the one measured in the imposed unloading situation from the very first series (Table 1, Fig 4A). Although the inhibition latency measured in the first series significantly differed from the latency found in the voluntary unloading situation, this difference was no longer present in the last series anymore (Table 1, Fig 4A).

The latency of the first inhibition characterizing the double inhibition pattern differed from the one reported during the voluntary unloading situation both for the first and the last series of the learning situation (Table 1, Fig 4B). Conversely, there was no difference between the latencies of the second inhibition in the first and last series and that measured in the imposed unloading situation (Table 1, Fig 4B).

#### Relation between the EMG pattern, inhibition onset and postural stabilization

**Fig 5:**
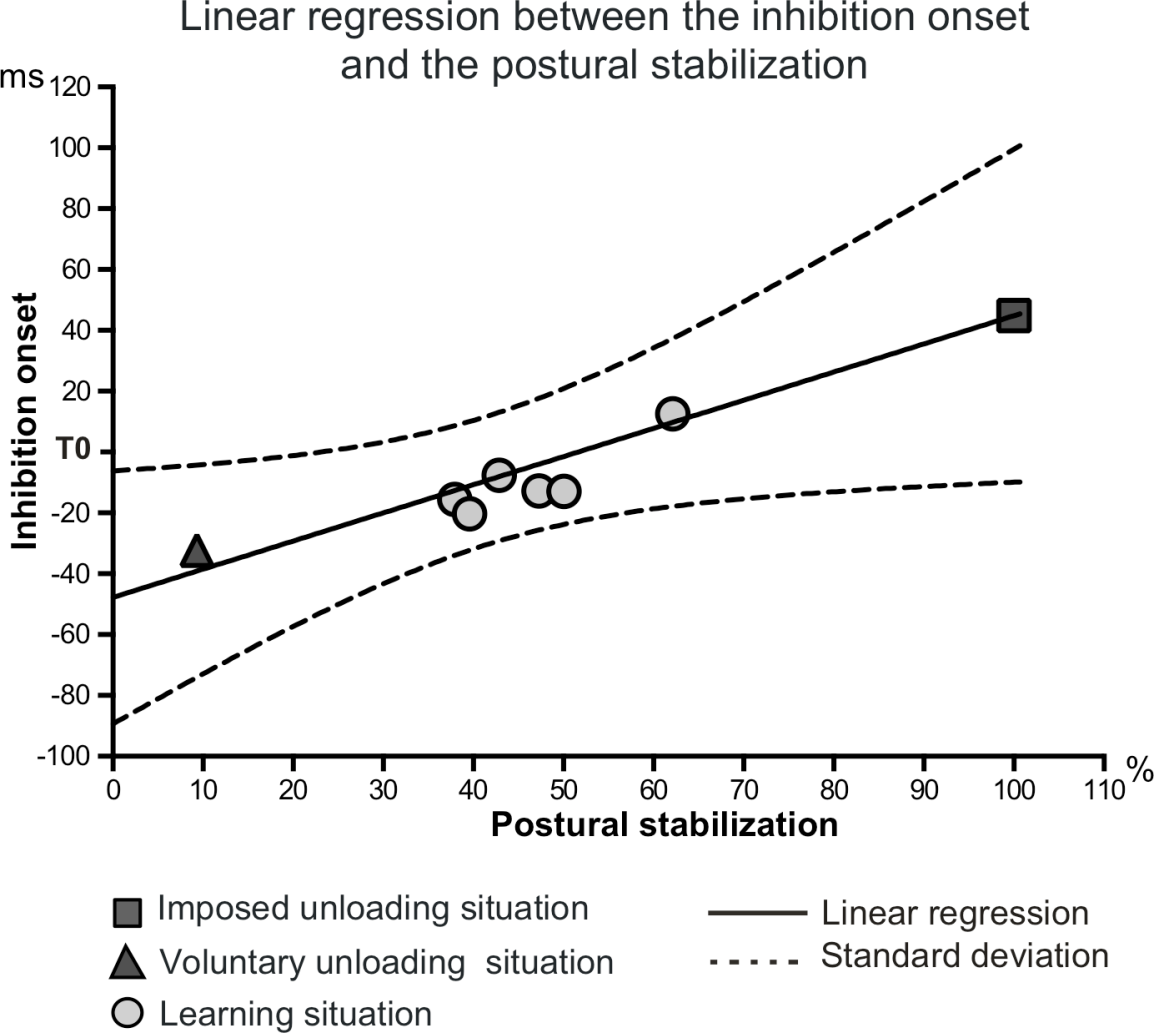
Linear regression between the onset of the single inhibition over the *biceps brachii* and the postural stabilization across the six series of the learning situation. The postural stabilization was quantified using the MA expressed in % of the imposed unloading situation. The lower the percentages, the better the stabilization. The mean inhibition latency and the mean MA% measured during the imposed unloading situation and the voluntary unloading situation, which were not entered in the regression analysis, have been reported on the same graph.

**1. Single inhibition pattern:** To uncover a functional link between the latency of the inhibition and the postural performance, a linear regression analysis was performed for each subject. At the individual level, a correlation analysis (Pearson correlation) between the inhibition onset and the MA% was first conducted. As it was significant for 9 subjects and near significance (p = 0.06) for 2 subjects, the linear regression analysis can be considered reliable. Moreover, the quantification of the distribution of the inhibition onsets enabled to verify that the values used in the linear regression were not pooled at two extremes (S2 Fig). Fig 5 presents the mean regression line between the latencies of the single inhibition over the *biceps brachii* and the MA% across the six learning series for the whole group. The slope of the mean regression line (0.936 ± 0.86) was significantly different from a theoretical zero value (*t*_(1,21)_ = 3.580, *p* = 0.005). We report the values of the mean latencies of the inhibition and the mean MA% measured during the imposed and the voluntary unloading situations on the same graph. The projection of these two values fell along the regression line.

**2. Double inhibition pattern:** The double inhibition pattern was characterized by a first inhibition that appeared earlier than the one found in the single inhibition pattern (*t*_(1,21)_ = 6.59, *p* < 0.001). We evaluated the postural consequences of this first inhibition by comparing the mean elbow rotation during that inhibition with the one measured during an equivalent period of time in the single inhibition pattern. To do so, we first defined a 50 ms-window starting from the mean latency of the first inhibition pattern. We also defined a 50 ms-window measured before any EMG events, i.e. -150 ms before the unloading onset, that we considered as a baseline time -window. We measured the mean elbow rotation in these two windows both in the single inhibition pattern and in the double inhibition pattern trials. During the baseline time -window, no difference in elbow rotation was found between the single and double inhibition patterns (-0.003 ± 0.11° for the single inhibition pattern; 0.012 ± 0.13° for the double inhibition pattern). However, there was a significant decrease in the mean elbow rotation measured during the first inhibition time window for the double inhibition pattern as compared to the single inhibition pattern (*U* = 17037; *p* < 0.001; 0.03 ± 0.18° for the single inhibition pattern; -0.17 ± 0.27° for the double inhibition pattern).

## Discussion

The main aim of this study was to identify a reliable neurophysiological marker reflecting the trial-by-trial acquisition of new APAs during learning. The EMG response underlying postural stabilization consists of an inhibition over the postural flexors, characterized by either a single or a double decrease in the EMG activity. Our main results showed that in the single inhibition pattern, the earlier the onset of the flexor inhibition, the better the postural stabilization whereas in the double inhibition one, the onset of the first inhibition occurred too early and consequently deteriorated the postural stabilization. This finding reveals that the precise timing of muscular inhibition response can be considered as a marker of the output of the learning of a new anticipatory postural control. Nevertheless, the great proportion of non-identifiable trials might limit further discussions. The non-identifiable trials at the end of the learning were present with a similar proportion to those found in the imposed and the voluntary unloading, suggesting that the proportion of non-identifiable EMG events decreases with practice. Additionally, no difference in the postural performance was found between the non-identifiable EMG pattern and the others. Lastly, a physiological account should be considered. Electromyographic signals translate a summation of the motor units of the muscle, which are synchronized spatially and temporally. At the beginning of the learning, the lack of temporally synchronized motor units might prevent the recording of a distinct EMG response. However, the fact that the motor unit activities might not be fully synchronized does not seem to prevent a good postural stabilization.

Learning can be defined as the modification of performance through practice. As modeled by the non-linear regression across the first learning series, postural performance in the learning situation underwent a fast modification during the first trials, followed by a slower improvement, thus indicating a rapid effect of practice. In the imposed unloading situation, the repetition of an unpredictable perturbation produces a slight decrease in the upward elbow displacement, probably due to a supra-spinal gain of the unloading reflex [3]. One might argue that the same process could explain the improvement of the postural performance occurring during learning. However, here the postural stabilization was largely improved as compared to the one obtained during the imposed unloading situation. This indicates that a learning process, which differs from the mere adaptation of the unloading reflex, took place.

### Searching for changes in the cocontraction pattern rate to track the learning process

Studies that have evaluated patterns of muscular activity in the early stages of learning report that after an initial phase of extensive use, a decrease in the cocontraction rate takes place as learning progresses [12,25]. In the bimanual load-lifting task, the cocontraction pattern is largely used by children to stabilize their forearm [4,5], then it disappears during adolescence [6]. In our study, the use of EMG recordings revealed that cocontractions are rarely present during learning. To understand why certain motor patterns are preferred to others, several studies place motor learning within an optimal control framework, in which a task is associated with a cost, such as the consumed energy [31]. As the cost of cocontractions is high in terms of muscular energy, their near absence in this task might indicate that learning does not rely on an optimization of the energy cost. Therefore, Changes in the rate of the cocontraction pattern could not be used to track the ongoing learning process.

### The shift of the flexor inhibition onset as a marker of the acquisition of new APAs

During the bimanual load-lifting task (i.e. the voluntary and the imposed unloading situations), the inhibition of the postural flexor reflects the electromyographic signature of the postural stabilisation but the two signatures differ by their latencies, implicating two kinds of processes sustaining postural control [2]. During imposed unloading, the unloading reflex appears after the load release, whereas during voluntary unloading, the early inhibition refers to the muscular expression of APAs. In the learning situation, we also found that the main pattern was one of inhibition. Concomitant with the learning of new APAs, its latency was smoothly altered in parallel with the improvement of the postural performance, therefore revealing a fine-tuning towards the optimized latency. In fact, a linear regression showed that the earlier the latency, the better the postural stabilization; thus implying a strong functional link between enhanced postural control and the accuracy of the progressive EMG timing. Interestingly, the values of the inhibition onset and the postural stabilization, measured during both imposed and voluntary unloading, were also along that straight regression line. These control situations somehow set the upper and lower limits of the timing of two kinds of inhibition, namely the unloading reflex during imposed unloading, and the acquired APAs during voluntary unloading, characterized by a late onset in the former case and an early one in the latter. This perfect superimposition along the learning regression line further evidence the shift from a reactive control into a predictive one. Interestingly, the progressive migration of the inhibition onset reveals a fine-tuning of the APAs towards an optimized latency across learning.

### The double inhibition pattern during learning: expression of concomitant predictive and reactive mode of control

Characterized by the presence of two inhibitions over the postural flexor, the double inhibition pattern could reflect the combination of two kinds of action control. Indeed, action control can be achieved either in a feedback manner, or in a feed-forward one. Feed-forward control occurs during a temporal window during which the control of action is made without the on-line use of sensory feedback information. Inversely, the feedback control is used to increase the action accuracy by providing an on-line movement correction [17]. The first inhibition, that starts well before the unloading onset, would be the electromyographic signature of a postural predictive control, whereas the second inhibition, that starts after the unloading onset, could rest on the on-line use of feedback information. To understand the functional role of the double inhibition pattern, we evaluated its effect on the horizontal position of the postural arm, which is considered the postural reference. The first inhibition, that appeared significantly earlier than the one found during voluntary unloading, resulted in a diminution of the elbow rotation before the load lifting; whereas within a similar temporal window, the forearm was still in a horizontal position in the single inhibition pattern. The exact timing of the inhibition onset is key to the efficiency of APAs [3]. Here, the first inhibition might have resulted from a premature feed-forward control that was launched too early, and consequently destabilized the reference position of the postural forearm before the lifting began. The latency of the second inhibition did not differ from the one found during imposed unloading, suggesting that the second inhibition might be similar to the unloading reflex that was measured in the imposed unloading situation. However, its postural consequences were dramatically different as it resulted in a reduced elbow rotation as compared to the one found in the imposed unloading situation. Further, the second inhibition of the double inhibition pattern occurred in a temporal window during which proprioceptive signals informing on the state of the musculoskeletal system are available [32]. Taken together, these elements suggest that the origin of the second inhibition in the double inhibition pattern arose from an on-line corrective mechanism based on a proprioceptive feedback loop.

### Learning of new APAs and the update of a forward predictive model

Computational work proposes that, depending on the task, learning can be maintained by the update of either the inverse or the forward model or both (see for review [9]). The inverse model is defined as a map that associates the goal of the movement (here, lifting a load) with motor commands that achieve that goal, whereas a forward model is a map that associates motor commands with their sensory consequences [33]. In the learning situation, where the task is to lift a load, during the first trial the inverse model implements the motor command by defining the spatio-temporal parameters of the lifting movement, without being able to predict its consequences on the forearm postural stabilization. As this first lift produces unpredicted sensory consequences on the postural forearm, a forward model has to be implemented to cancel out the unexpected shortening of the musculoskeletal fibers of the postural flexors, which in turns modifies the horizontal postural reference. By capturing the causal relationship between the action and its postural outcome, the forward model progressively refines the sensory prediction of the consequences of the load lifting on the postural control. Because it predicts the consequences of the motor command on the postural control before the action starts, here, learning relied on the update of a forward predictive model. The signal used to update a model depends on the corrective error signal provided by the comparison between the actual sensory feedback and the predicted sensory feedback [9,34]. When shortened, the muscle spindles of the flexors generate a proprioceptive feedback, which is then used to calculate the corrective error signal by comparison to the predicted sensory feedback. Maximal during the first trial, then diminishing with learning, the corrective error signal is used to update the forward predictive model. Importantly, our study suggests that the output of the update of the forward predictive model might be the inhibition onset that cancels out the sudden shortening of the musculoskeletal fibers.

## Conclusion

To conclude, our study reveals a progressive shift towards an earlier onset of the inhibition over the postural flexor across learning. The accurate timing of the flexor inhibition could constitute a reliable neurophysiological marker of the on-line acquisition of new APAs. Timing is affected in neurological illness such as Parkinson’s disease [35] and also in neurodevelopmental disorders, such as autism [36] and developmental coordination disorders [37]. Therefore, the possibility to track an on-going learning process with such high temporal precision could be used to explore possible dysfunctions in motor learning processes in degenerative and neurodevelopmental pathologies.

## Supporting Information

S1 Fig Distribution of the EMG pattern through subjects. The number of trials was reported for each subject during each series of the learning situation: for (A) the non-identifiable trials, (B) the single inhibition pattern and (C) the double inhibition pattern.
(TIFF)

S2 Fig Distribution of the inhibition onset values across trials in the learning situation. The number of trials was reported across a window starting at -115ms and ending at +78ms, with a 2-ms step. The limits (mean ± standard deviation) of the time-windows where the EMG responses occurred in the voluntary and imposed unloading situations are also indicated.
(TIFF)
