# Supplementary figures and images for: Shift of the Muscular Inhibition Latency during On-Line Acquisition of Anticipatory Postural Adjustments
Source: PLoS One. 2016 May 18;11(5):e0154775. doi: 10.1371/journal.pone.0154775 (PMC4871431; doi:10.1371/journal.pone.0154775)

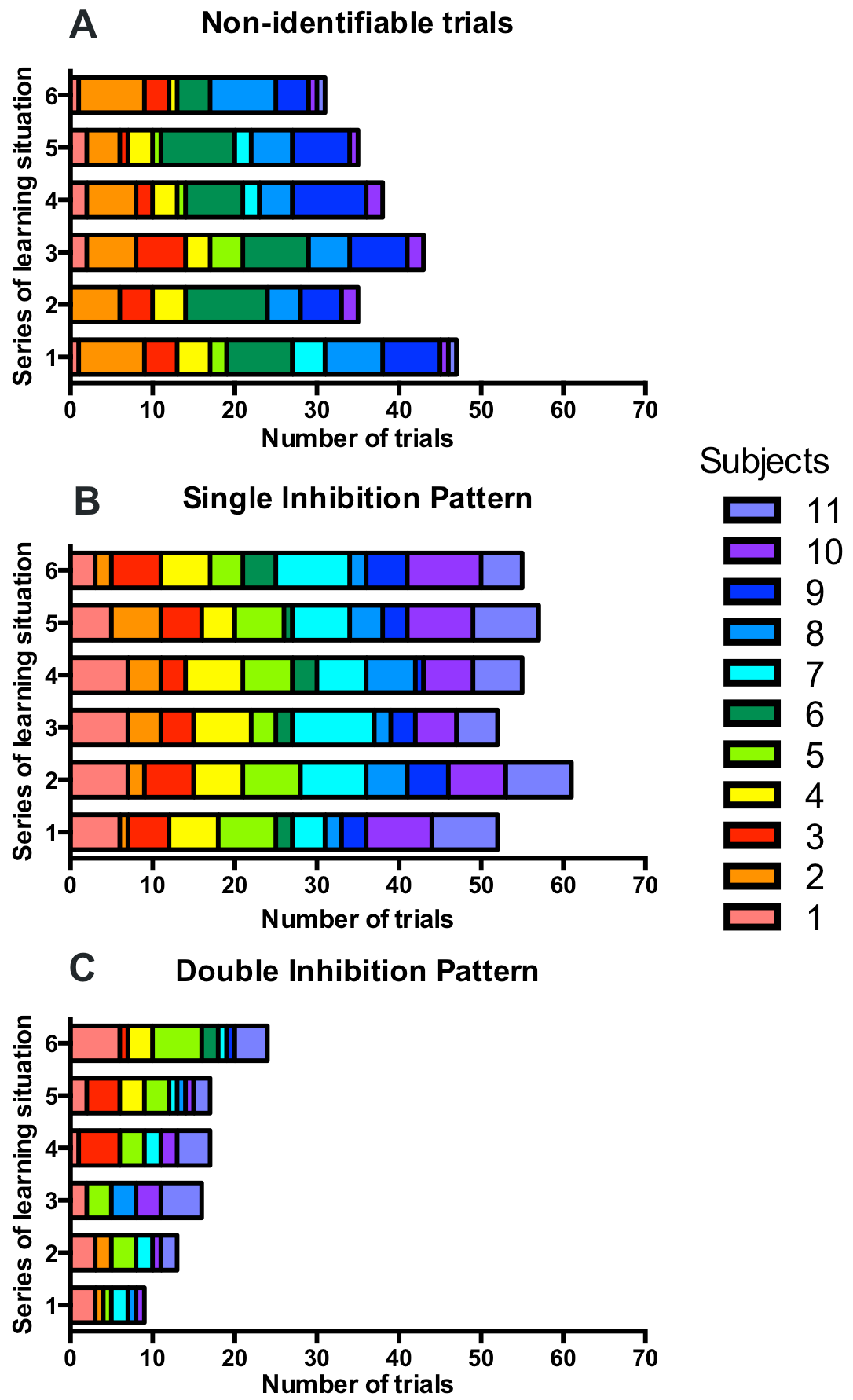

Supplement: S1 Fig — The number of trials was reported for each subject during each series of the learning situation: for (A) the non-identifiable trials, (B) the single inhibition pattern and (C) the double inhibition pattern. (TIFF) [file pone.0154775.s001.tiff]

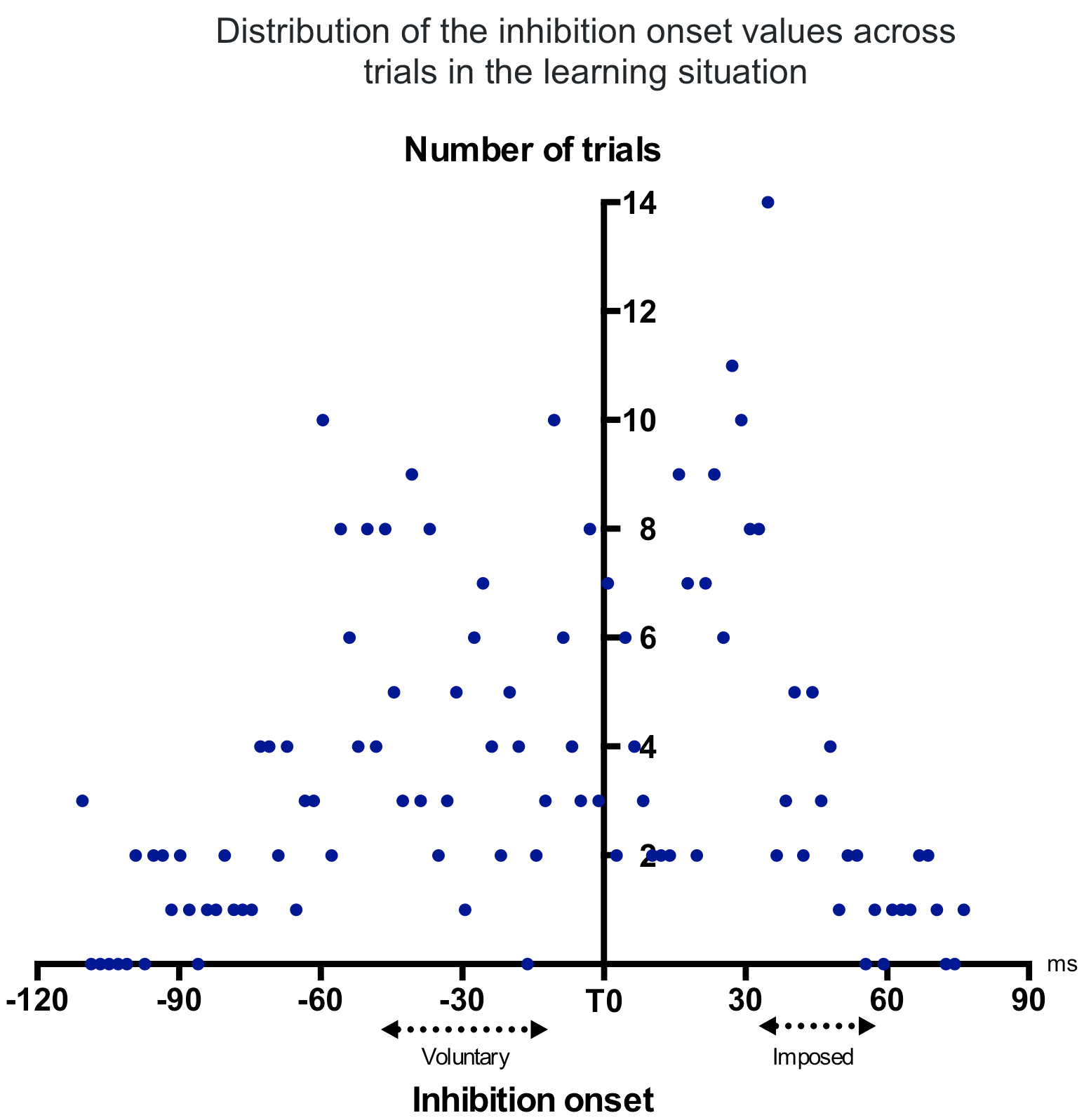

Supplement: S2 Fig — The number of trials was reported across a window starting at -115ms and ending at +78ms, with a 2-ms step. The limits (mean ± standard deviation) of the time-windows where the EMG responses occurred in the voluntary and imposed unloading situations are also indicated. (TIFF) [file pone.0154775.s002.tiff]
